# Supplementary material for: The homeodomain transcriptional regulator DVE-1 directs a program for synapse elimination during circuit remodeling
Source: Nat Commun. 2023 Nov 18;14:7520. doi: 10.1038/s41467-023-43281-4 (PMC10657367; doi:10.1038/s41467-023-43281-4)
Supplement: Supplementary file 1 — Supplementary Information [file 41467_2023_43281_MOESM1_ESM.pdf]

The homeodomain transcriptional regulator DVE-1 directs a program for synapse elimination during circuit remodeling

Supplemental Figures

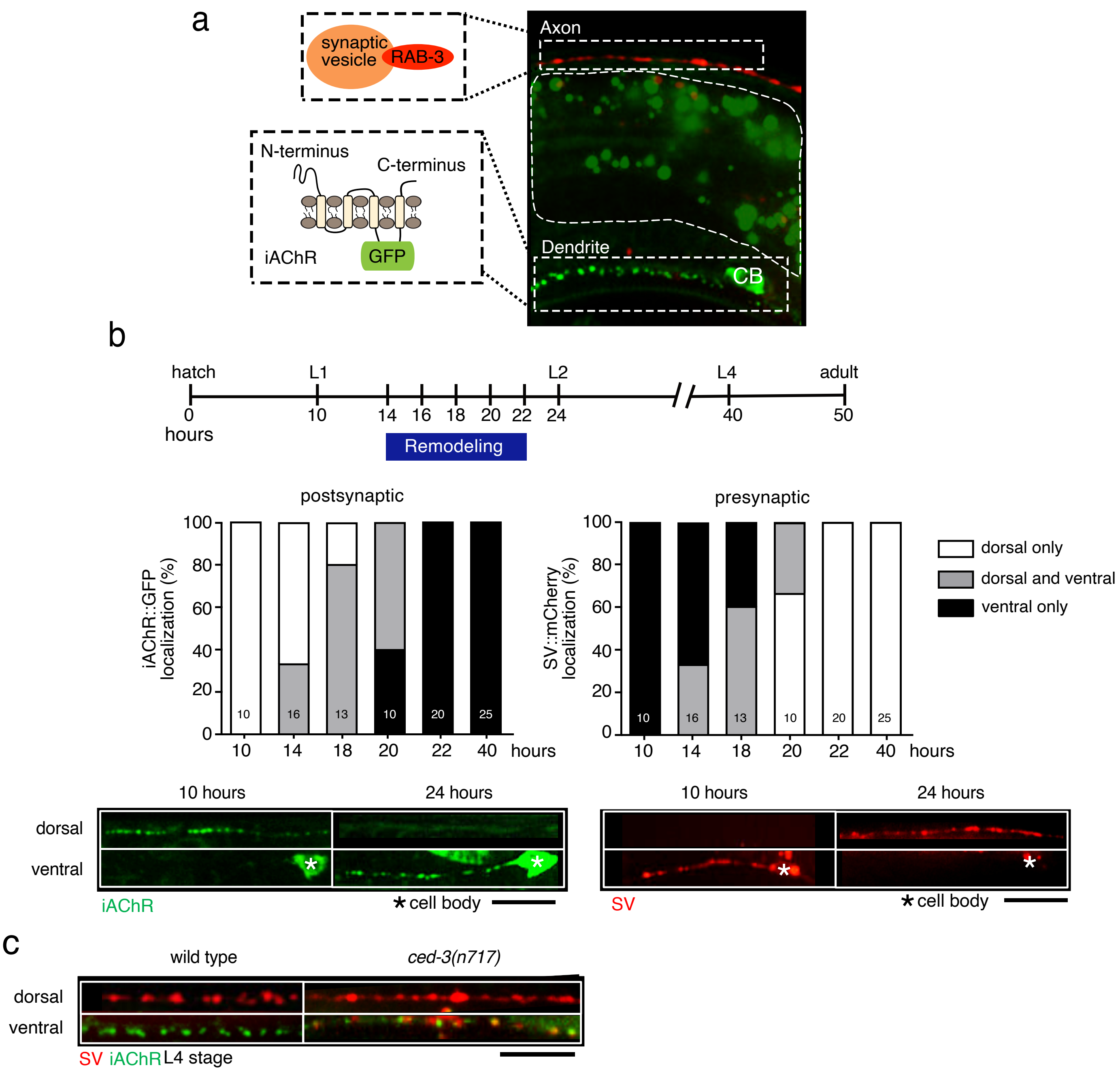

Supplemental Figure 1.1. Remodeling of cholinergic postsynaptic sites and GABAergic presynaptic terminals occur simultaneously but are regulated through different mechanisms

(A) Schematics and confocal image of L4 stage wild type animal co-expressing the synaptic vesicle marker mCherry::RAB-3 with the iAChR marker ACR-12::GFP in DD neurons. Insets are schematics of each marker. Masking of intestinal autofluorescence is outlined by white dotted line.

(B) Top, timeline of wild type development. Approximate timing of transitions between larval stages and to adulthood are indicated. Blue bar indicates duration of DD synaptic remodeling in wild type animals. Middle, quantification of iAChR (postsynaptic) remodeling (left) and SV (presynaptic) remodeling in DD neurons (right) at the indicated time points after hatch. Animals are binned as dorsal only (white), ventral only (black), or dorsal and ventral (grey) according to the distribution of iAChR (left) or SV (right) clusters. Bottom, representative images of dorsal and ventral iAChR clusters (left) and SV puncta (right) at the times indicated in DD neurons of wild type animals. Remodeling of iAChR clusters and SV puncta occur simultaneously. Scale bar, 5 μm.

(C) Merged confocal images showing SV puncta (red) and iAChR clusters (green) in dorsal and ventral DD neuron processes of L4 stage wild type (left) and *ced-3(n717)* mutants (right). Scale bar, 5 μm.

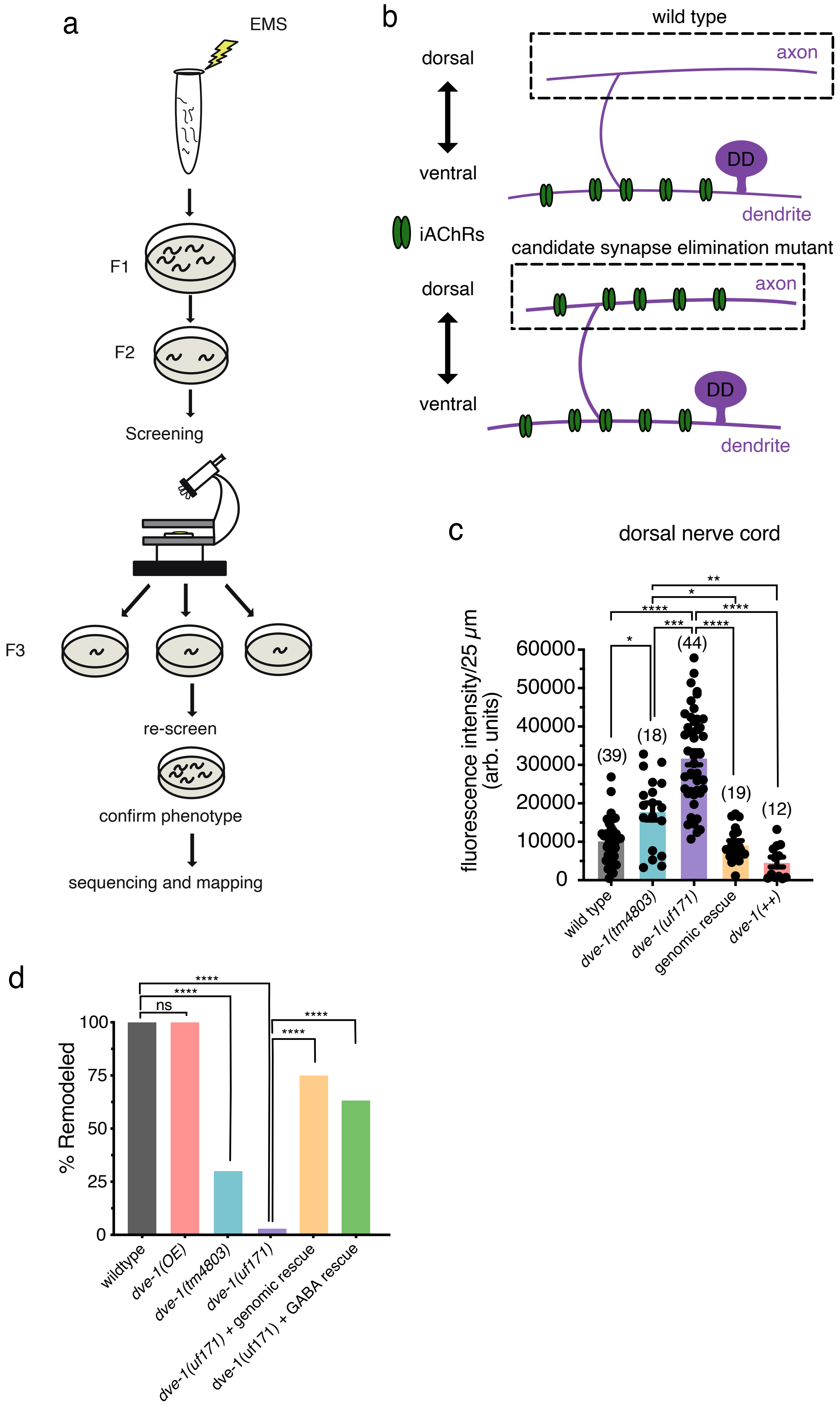
a
EMS
F1
F2
Screening
F3
re-screen
confirm phenotype
sequencing and mapping
b
wild type
dorsal
axon
ventral
DD
dendrite
iAChRs
candidate synapse elimination mutant
dorsal
axon
ventral
DD
dendrite
c
dorsal nerve cord
fluorescence intensity/25 μm (arb. units)
60000
50000
40000
30000
20000
10000
0
(39)
(18)
(44)
(19)
(12)
wild type
dve-1(tm4803)
dve-1(uf171)
genomic rescue
dve-1(++)
d
% Remodeled
100
75
50
25
0
ns
wildtype
dve-1(OE)
dve-1(tm4803)
dve-1(uf171)
dve-1(uf171) + genomic rescue
dve-1(uf171) + GABA rescue

Supplemental Figure 1.2. A Forward genetic screen to identify conserved mechanisms controlling synapse elimination

(A) Schematic of experimental workflow for ethyl methanesulfonate (EMS) screen to obtain mutants with defects in the elimination of juvenile dorsal iAChR clusters.

(B) Schematics of iAChR localization within DD neurons of L4 stage wild type (left) or potential synapse elimination mutant (right).

(C) Quantification of the average ACR-12::GFP fluorescence intensity per 25 μm in the dorsal nerve cord. Bars indicate mean ± SEM. Only significant comparisons shown, ****$p<0.0001$, ***$p<0.001$, **$p<0.01$, *$p<0.05$, one-way ANOVA with tukey's multiple comparisons test. Each point represents a single animal.

(D) The percentage of L4 stage animals where dorsal iAChRs have been completely removed. ****$p<0.0001$, two-tailed Fischer's exact test with Bonferroni Correction. Complete remodeling: ventral AChRs only. Incomplete remodeling: dorsal and ventral AChRs.

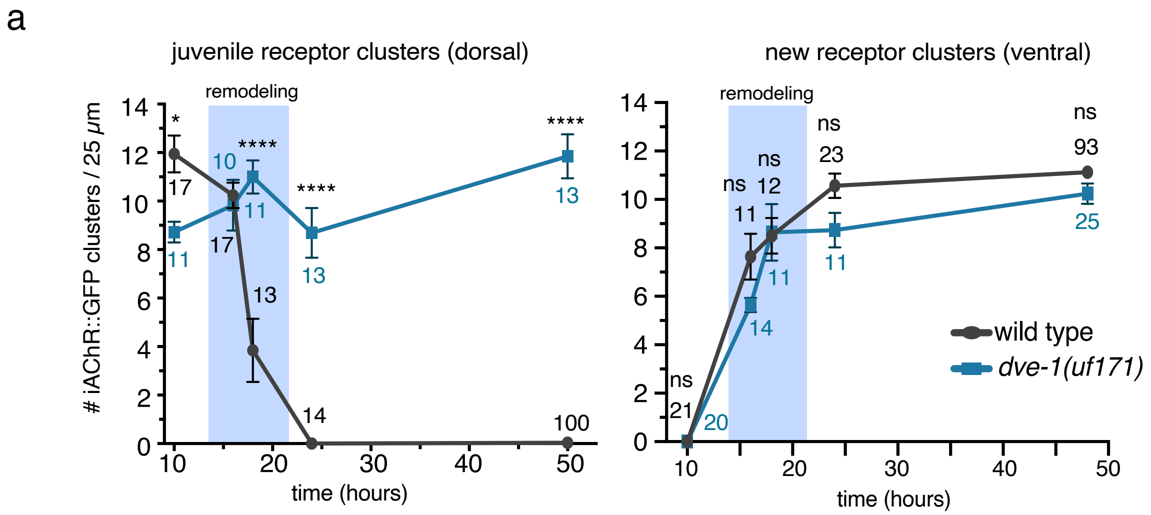

a
juvenile receptor clusters (dorsal)
new receptor clusters (ventral)
remodeling
iAChR::GFP clusters / 25 μm
time (hours)
wild type
dve-1(uf171)

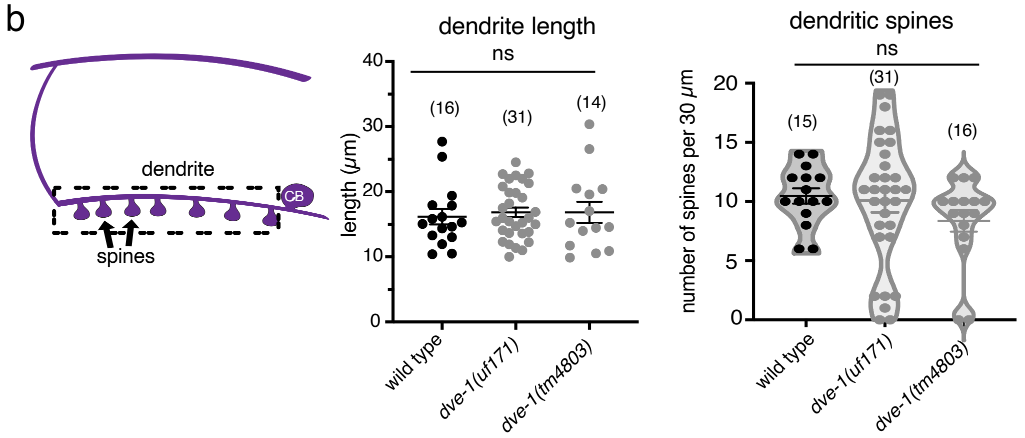

b
dendrite
CB
spines
dendrite length
ns
length (μm)
(16)
(31)
(14)
wild type
dve-1(uf171)
dve-1(tm4803)
dendritic spines
number of spines per 30 μm
(15)
(31)
(16)

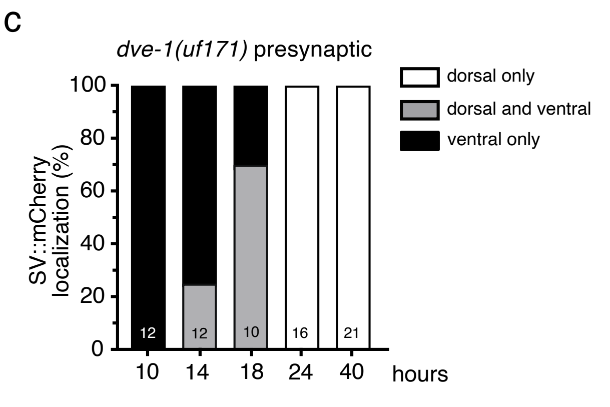

c
dve-1(uf171) presynaptic
SV::mCherry localization (%)
dorsal only
dorsal and ventral
ventral only
hours

Supplemental Figure 2.1. *dve-1* mutation disrupts synapse elimination but does not affect new synapse formation

(A) Average number of juvenile iAChR clusters in the dorsal nerve cord (left) and new iAChR clusters in the ventral nerve cord (right) at the indicated times after hatch. iAChR clusters are removed from the dorsal nerve cord of wild type animals (black) during remodeling (blue shading) but persist in in the dorsal nerve cord of *dve-1* mutants (blue). iAChR clusters in the ventral nerve cords of wild type and *dve-1* mutants increase similarly over time. Data points indicate mean ± SEM. ****p<0.0001, two-tailed student's t-test.

(B) Far left: Schematic of DD neuron, segmented box represents area quantified in D. Arrows indicate dendritic spines. CB, cell body. Scatterplots of average length of DD neuron dendrite (left) and number of dendritic spines (right) in wild type, *dve-1(uf171)*, *dve-1(tm4803)* overlayed with a violin plot to show distribution. Each point represents a single animal. Line represents mean ± SEM. ns: not significant.

(C) Quantification of GABA synaptic vesicle (mCherry::RAB-3) remodeling in DD neurons at the indicated time points after hatch in *dve-1(uf171)* mutant animals. Animals are binned as dorsal only (white), ventral only (black), or dorsal and ventral (grey) according to the distribution of SV clusters. See S1.1B for wild type comparison.

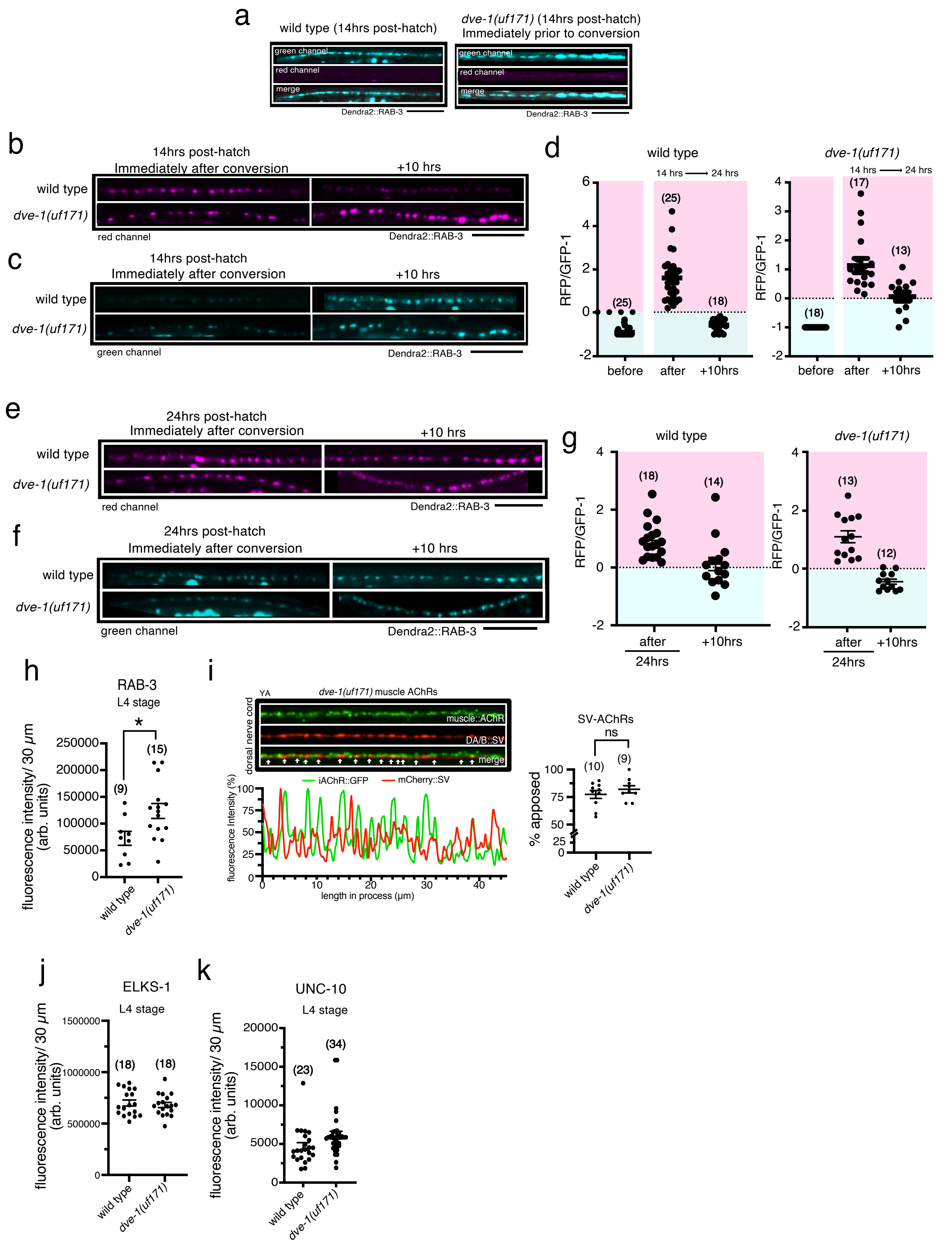
a
wild type (14hrs post-hatch)
dve-1(uf171) (14hrs post-hatch)
Immediately prior to conversion
green channel
red channel
merge
Dendra2::RAB-3
b
14hrs post-hatch
Immediately after conversion
+10 hrs
wild type
dve-1(uf171)
red channel
Dendra2::RAB-3
c
14hrs post-hatch
Immediately after conversion
+10 hrs
wild type
dve-1(uf171)
green channel
Dendra2::RAB-3
d
wild type
dve-1(uf171)
14 hrs → 24 hrs
RFP/GFP-1
(25)
(25)
(18)
(18)
(17)
(13)
before
after
+10hrs
e
24hrs post-hatch
Immediately after conversion
+10 hrs
wild type
dve-1(uf171)
red channel
Dendra2::RAB-3
f
24hrs post-hatch
Immediately after conversion
+10 hrs
wild type
dve-1(uf171)
green channel
Dendra2::RAB-3
g
wild type
dve-1(uf171)
RFP/GFP-1
(18)
(14)
(13)
(12)
after
+10hrs
24hrs
h
RAB-3
L4 stage
*
(9)
(15)
fluorescence intensity/ 30 μm
(arb. units)
wild type
dve-1(uf171)
i
YA
dve-1(uf171) muscle AChRs
dorsal nerve cord
muscle::AChR
DA/B::SV
merge
iAChR::GFP
mCherry::SV
fluorescence Intensity (%)
length in process (μm)
SV-AChRs
ns
(10)
(9)
% apposed
wild type
dve-1(uf171)
j
ELKS-1
L4 stage
(18)
(18)
fluorescence intensity/ 30 μm
(arb. units)
wild type
dve-1(uf171)
k
UNC-10
L4 stage
(23)
(34)
fluorescence intensity/ 30 μm
(arb. units)
wild type
dve-1(uf171)

Supplemental Figure 3.1. Presynaptic cholinergic synaptic vesicles turnover during remodeling

(A) Dendra2::RAB-3 clusters in the dorsal nerve cord of wild type (left) and *dve-1(uf171)* mutants (right) prior to photoconversion.

(B) Cholinergic Dendra2::RAB-3 clusters (red channel) in the dorsal nerve cord (*unc-129pr*::Dendra2::RAB-3) of wild type (top) and *dve-1(uf171)* mutants (bottom) either immediately after green to red photoconversion at 14 hours after hatch (left) or 10 hours later (right). Juvenile Dendra2::RAB-3 clusters are largely removed during wild type remodeling but are more stable in *dve-1* mutants. Scale bar, 5 μm.

(C) Cholinergic Dendra2::RAB-3 clusters (green channel) in the dorsal nerve cord of wild type (top) and *dve-1(uf171)* mutants (bottom) either immediately after green to red photoconversion at 14 hours after hatch (left) or 10 hours later (right). Green Dendra2::RAB-3 clusters are similarly added during remodeling of wild type and *dve-1* mutants. Scale bar, 5 μm.

(D) Scatterplots of Dendra2::RAB-3 RFP/GFP fluorescence intensity ratios before photoconversion at 10 hours after hatch (before remodeling), immediately after, and 10 hours later for wild type and *dve-1* mutants. Expressed as RFP/GFP fluorescence ratio -1 for display purposes. Negative values indicate enhanced green fluorescence while positive values enhanced red fluorescence. Each dot represents a single animal.

(E) Cholinergic Dendra2::RAB-3 clusters (red channel) in the dorsal nerve cord of wild type (top) and *dve-1(uf171)* mutants (bottom) either immediately after green to red photoconversion at 24 hours after hatch (left) or 10 hours later (right). For both wild type and *dve-1*, photoconverted cholinergic Dendra2::RAB-3 clusters are stable following remodeling. Scale bar, 5 μm.

(F) Cholinergic Dendra2::RAB-3 clusters (green channel) in the dorsal nerve cord of wild type (top) and *dve-1(uf171)* mutants (bottom) either immediately after green to red photoconversion at 24 hours after hatch (left) or 10 hours later (right). Scale bar, 5 μm.

(G) Scatterplots of Dendra2-RAB-3 RFP/GFP fluorescence intensity ratios before photoconversion at 24 hours after hatch (following remodeling), immediately after, and 10 hours later for wild type and *dve-1* mutants. Expressed as RFP/GFP fluorescence ratio-1 for display purposes. Negative values indicate enhanced green fluorescence while positive values indicate enhanced red fluorescence. Each dot represents a single animal.

(H) Scatterplot of cholinergic mCherry::RAB-3 fluorescence intensity (*unc-129pr*::mCherry::RAB-3) in L4 stage dorsal nerve cord (DNC) for wild type and *dve-1* mutants. Each point indicates a single animal. Bars indicate mean ± SEM. *$p<0.01$, two-tailed student's t-test.

(I) Top, apposition of dorsal muscle iAChRs (*myo-3pr*::ACR-16::GFP, green) and cholinergic synaptic vesicles (SV, *acr-5pr*::mCherry::RAB-3, red) in the dorsal nerve cord of L4 stage *dve-1* mutant. Bottom, line scan of relative muscle iAChR (green) and cholinergic SV (red) fluorescence intensity for the same 44 μm region. Right, percent apposition between muscle iAChR and cholinergic SV clusters for wild type and *dve-1(uf171*) mutants. Each point represents a single animal. Bars indicate mean ± SEM. ns: not significant, two-tailed student's t-test.

(J) Scatterplot of cholinergic ELKS-1::mCherry fluorescence intensity (*unc-129pr*::ELKS-1::mCherry) in L4 stage dorsal nerve cord (DNC) for wild type and *dve-1* mutants. Each point indicates a single animal. Bars indicate mean ± SEM.

(K) Scatterplot of cholinergic UNC-10::GFP fluorescence intensity (*acr-5pr*::UNC-10::GFP) in L4 stage dorsal nerve cord (DNC) for wild type and *dve-1* mutants. Each point indicates a single animal. Bars indicate mean ± SEM.

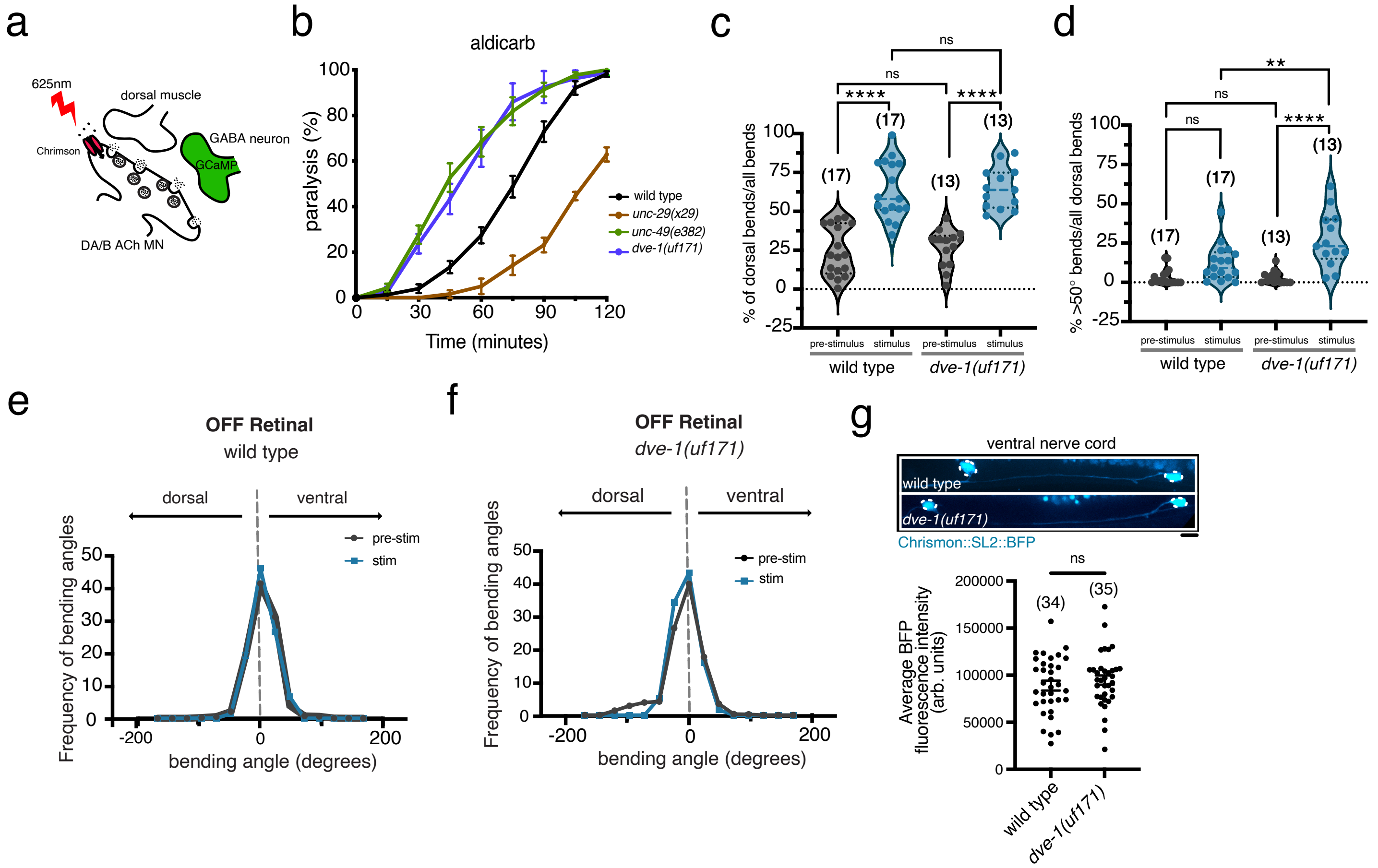
a
625nm
dorsal muscle
Chrimson
GABA neuron
GCaMP
DA/B ACh MN
b
aldicarb
paralysis (%)
Time (minutes)
wild type
unc-29(x29)
unc-49(e382)
dve-1(uf171)
c
% of dorsal bends/all bends
ns
ns
****
****
(17)
(17)
(13)
(13)
pre-stimulus
stimulus
wild type
dve-1(uf171)
d
% >50° bends/all dorsal bends
**
ns
ns
****
(17)
(17)
(13)
(13)
pre-stimulus
stimulus
wild type
dve-1(uf171)
e
OFF Retinal
wild type
dorsal
ventral
Frequency of bending angles
pre-stim
stim
bending angle (degrees)
f
OFF Retinal
dve-1(uf171)
dorsal
ventral
Frequency of bending angles
pre-stim
stim
bending angle (degrees)
g
ventral nerve cord
wild type
dve-1(uf171)
Chrismon::SL2::BFP
ns
(34)
(35)
Average BFP fluorescence intensity (arb. units)
wild type
dve-1(uf171)

Supplemental Figure 4.1. Motor circuit function is disrupted in *dve-1* mutants

(A) Schematic of combined cell-specific expression of Chrimson (red receptor) for cholinergic depolarization, and GCaMP6s monitoring [Ca^{2+}] changes in the post-synaptic GABAergic motor neurons (green).

(B) Time course of paralysis in the presence of aldicarb (1 mM) for wild type (black) (n=14), *unc-29(x29)* mutants (brown) (n=6), *unc-49(e382)* mutants (dark green) (n=16), *dve-1(uf171)* mutants (blue) (n=12), are shown. At least 10 animals per trial. Data represent mean ± SEM.

(C) Scatterplot with violin overlay of the percentage of dorsal bends for wild type and *dve-1(uf171)* mutants before and after photostimulation. Each point represents a single animal. *****p*<0.0001, ns: not significant, two way ANOVA with Tukey's multiple comparisons test.

(D) Scatterplot with violin overlay of the percentage of dorsal turns greater than 50° wild type (black) and *dve-1(uf171)* (blue) before and after photostimulation. Each point represents a single animal. *****p*<0.0001, ***p*<0.01, ns - not significant, two way ANOVA with Tukey's multiple comparisons test.

(E) Frequency distribution of body bending angles prior to (black) and during photostimulation (blue) for control animals in the absence of all-trans-retinal. Negative bending angle values indicate dorsal, while positive bending angle values indicate ventral. n=3.

(F) Frequency distribution of body bending angles prior to (black) and during photostimulation (blue) for *dve-1* mutants in the absence of all-trans-retinal. Negative bending angle values indicate dorsal, while positive bending angle values indicate ventral. n=3.

(G) Top, confocal images of DA/DB motor neurons from control and *dve-1(uf171)* mutant animals expressing *punc-129::*Chrimson*::SL2::BFP*. Scale bar, 5 µm. Bottom, average fluorescence intensity of DA/DB neuron cell bodies labeled by *punc-129::*Chrimson*::SL2::BFP*. Each dot represents a single DA/DB cell body, at least 15 animals per genotype were imaged.

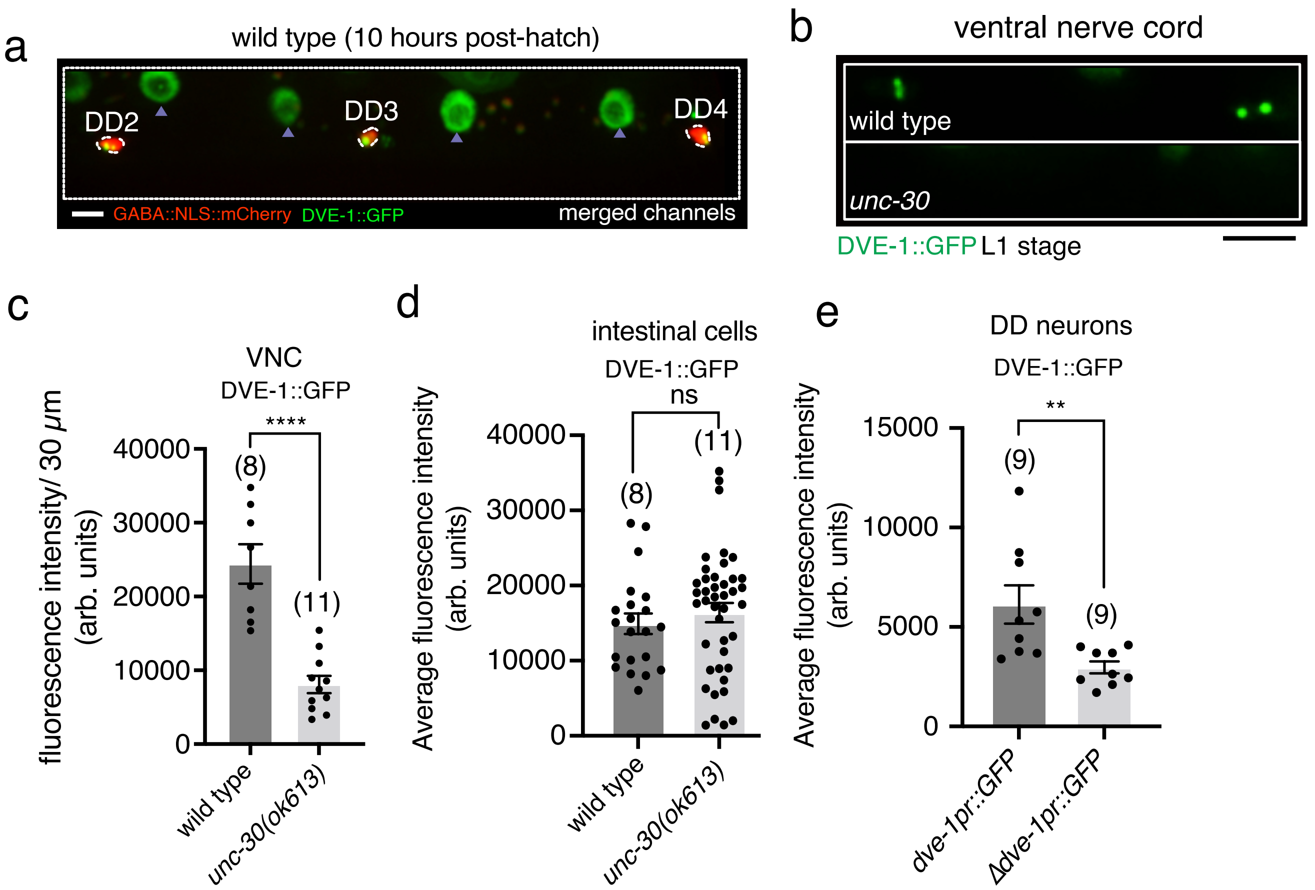
a
wild type (10 hours post-hatch)
DD2
DD3
DD4
GABA::NLS::mCherry
DVE-1::GFP
merged channels
b
ventral nerve cord
wild type
unc-30
DVE-1::GFP
L1 stage
c
VNC
DVE-1::GFP
****
fluorescence intensity/ 30 μm (arb. units)
(8)
(11)
wild type
unc-30(ok613)
d
intestinal cells
DVE-1::GFP
ns
Average fluorescence intensity (arb. units)
(8)
(11)
wild type
unc-30(ok613)
e
DD neurons
DVE-1::GFP
**
Average fluorescence intensity (arb. units)
(9)
(9)
dve-1pr::GFP
Δdve-1pr::GFP

Supplemental Figure 6.1. The Pitx family transcription factor UNC-30 regulates expression of DVE-1 in GABAergic neurons

(A) Ventral nerve cord expression of DVE-1::GFP in DD GABAergic motor neurons (white outlines) of L1 stage wild type animals. Blue arrowheads indicate intestinal cells. Scale bar, 5 μm.

(B) Images of ventral nerve cord DVE-1::GFP expression in DD GABAergic motor neurons of L1 stage wild type and *unc-30(ok613)* mutants. Scale bar, 5 μm.

(C) Scatterplot of average DVE-1::GFP fluorescence intensity in 30 μm region of ventral nerve cord. Each dot represents a single animal. Bars indicate mean ± SEM. ****p<0.0001, two-tailed student's t-test.

(D) Scatterplot of average DVE-1::GFP fluorescence intensity in intestinal cells of L1 stage wild type and *unc-30(ok613)* mutants. Each point represents a single intestinal cell. Imaged 3 intestinal cells per animal. Bars indicate mean ± SEM. ns: not significant, two-tailed student's t-test.

(E) Scatterplot of average nuclear GFP fluorescence intensity in DD neurons of L1 stage animals expressing either DVE-1::GFP using either native ~5 kb *dve-1* promoter region [*dve-1pr::DVE-1::GFP*] or the same promoter region lacking putative UNC-30 binding sites [*∆dve-1pr::DVE-1::GFP*]. Each point represents a single DD1 neuron from a different animal. Bars indicate mean ± SEM. **p<0.001, two-tailed student's t-test.

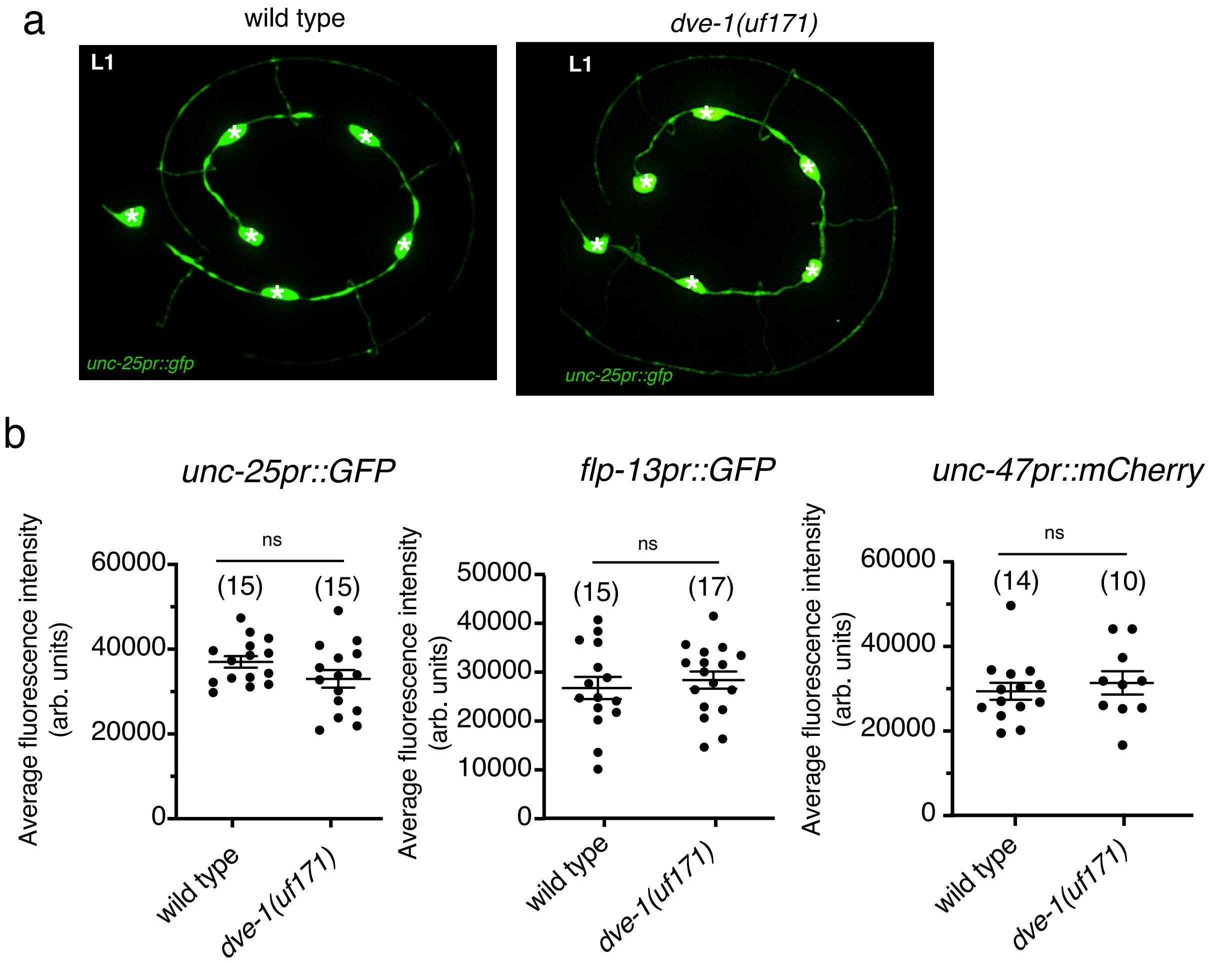

a
wild type
dve-1(uf171)
L1
L1
unc-25pr::gfp
unc-25pr::gfp
b
unc-25pr::GFP
flp-13pr::GFP
unc-47pr::mCherry
Average fluorescence intensity (arb. units)
ns
ns
ns
(15)
(15)
(15)
(17)
(14)
(10)
60000
40000
20000
0
50000
40000
30000
20000
10000
0
60000
40000
20000
0
wild type
dve-1(uf171)
wild type
dve-1(uf171)
wild type
dve-1(uf171)

Supplemental Figure 6.2. Mutation of *dve-1* does not alter GABAergic neuronal identity

(A) Representative image of wild type (right) and *dve-1(uf171)* mutant (left) animals expressing *punc-25::GFP* to label DD neurons at the L1 stage. * indicates cell body.

(B) Average fluorescence intensity of *unc-47pr::mCherry,* and *unc-25pr::GFP, flp-13pr::GFP* reporters in DD1, DD2, and DD3 neuron cell bodies of L1 stage wild type and *dve-1(uf171)* mutants. Each dot represents the average of three cell bodies in a single animal. Bars indicate mean ± SEM. n: not significant, two-tailed students t-test.

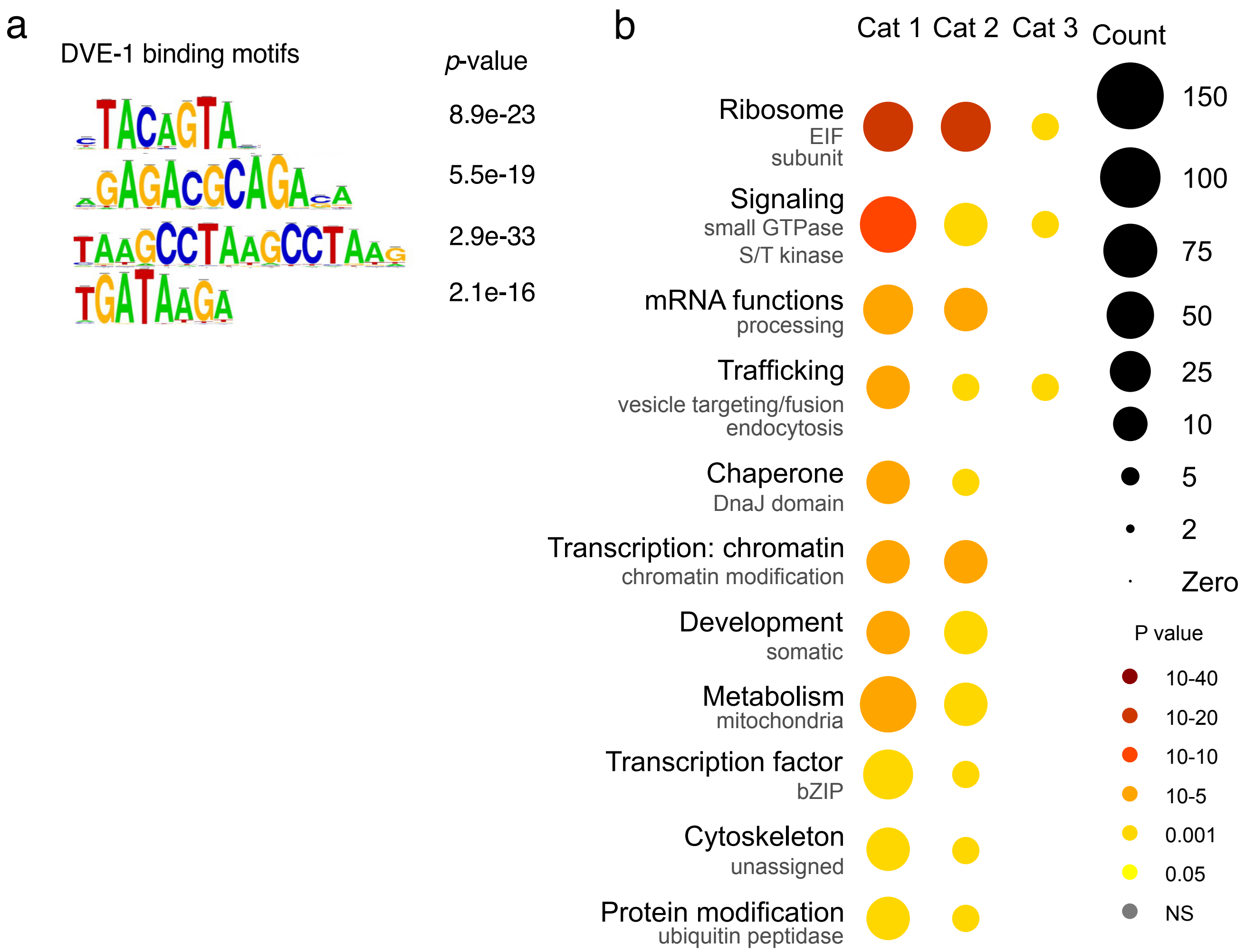
a
DVE-1 binding motifs
p-value
TACAGTA
8.9e-23
GAGACGCAGA
5.5e-19
TAAGCCTAAGCCTAA
2.9e-33
TGATAAGA
2.1e-16
b
Cat 1
Cat 2
Cat 3
Count
Ribosome
EIF
subunit
Signaling
small GTPase
S/T kinase
mRNA functions
processing
Trafficking
vesicle targeting/fusion
endocytosis
Chaperone
DnaJ domain
Transcription: chromatin
chromatin modification
Development
somatic
Metabolism
mitochondria
Transcription factor
bZIP
Cytoskeleton
unassigned
Protein modification
ubiquitin peptidase
150
100
75
50
25
10
5
2
Zero
P value
10-40
10-20
10-10
10-5
0.001
0.05
NS

Supplemental Figure 7.1. Enrichment analysis of putative DVE-1 targets reveals potential pathways governing synapse elimination

(A) *de novo* motif discovery analysis of 968 DVE-1 binding peaks identifies 4 DVE-1 binding motifs.

(B) Wormcat analysis for enriched categories of putative DVE-1 targets identified from ChIP-seq dataset. Size of circles indicates the number of genes and color indicates the significance value for over-representation in each Wormcat category.

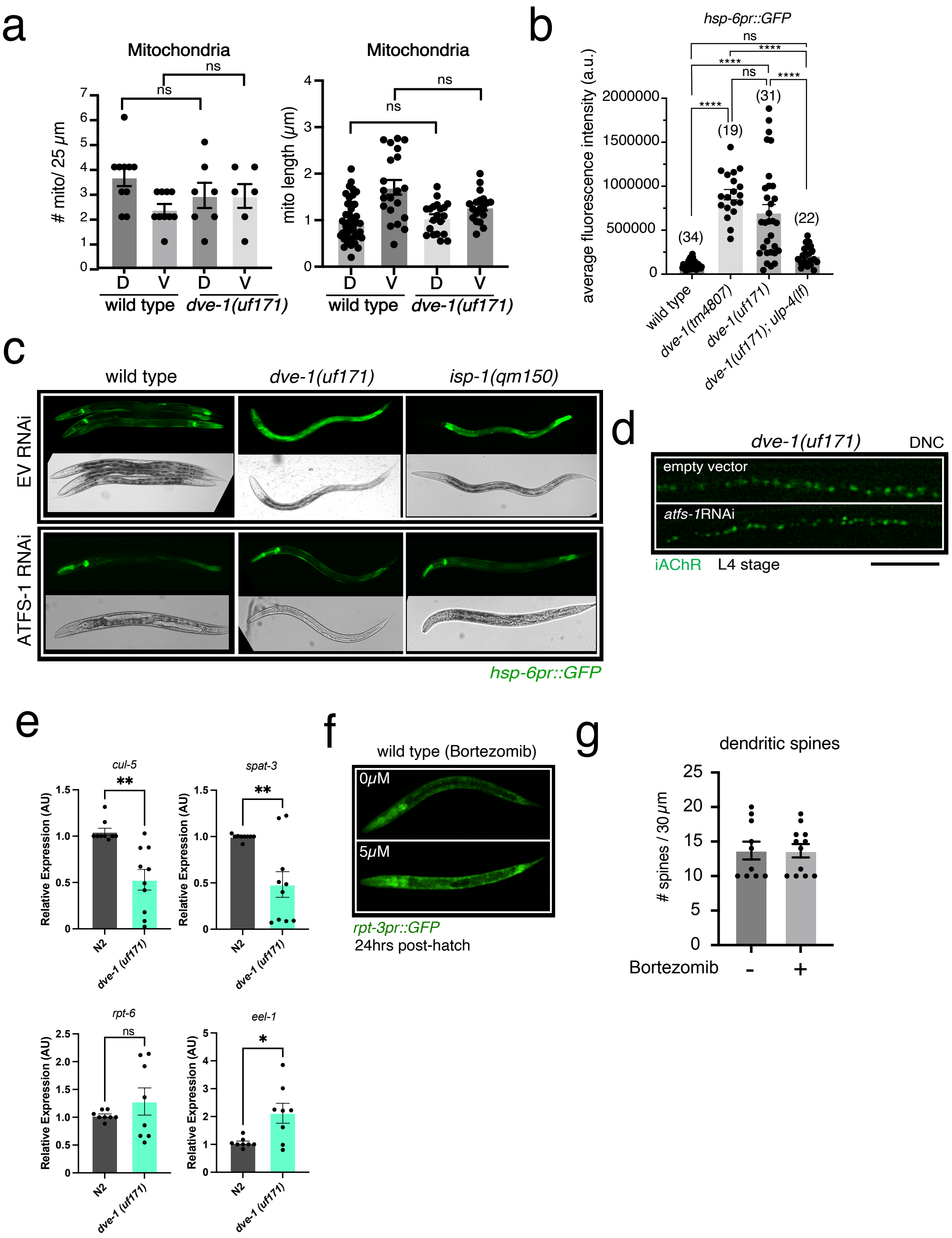

a
Mitochondria
mito/ 25 μm
ns
ns
D V D V
wild type dve-1(uf171)
Mitochondria
mito length (μm)
ns
ns
D V D V
wild type dve-1(uf171)
b
hsp-6pr::GFP
average fluorescence intensity (a.u.)
ns
****
****
ns
****
****
(34) (19) (31) (22)
wild type
dve-1(tm4807)
dve-1(uf171)
dve-1(uf171); ulp-4(lf)
c
wild type
dve-1(uf171)
isp-1(qm150)
EV RNAi
ATFS-1 RNAi
hsp-6pr::GFP
d
dve-1(uf171)
DNC
empty vector
atfs-1RNAi
iAChR
L4 stage
e
cul-5
**
Relative Expression (AU)
N2
dve-1 (uf171)
spat-3
**
rpt-6
ns
eel-1
*
f
wild type (Bortezomib)
0μM
5μM
rpt-3pr::GFP
24hrs post-hatch
g
dendritic spines
spines / 30 μm
Bortezomib
-
+

Supplemental Figure 7.2. Removal of iAChRs in GABA motor neurons is not affected by activation or inhibition of the mtUPR

(A) Left, scatterplot of mitochondrial density (number of mitochondria/25 μm) in dorsal (D) and ventral (V) processes of DD neurons of L4 stage wild type and *dve-1(uf171)* mutants. Each point represents a single animal. wild type, n=10; *dve-1(uf171)*, n=6. Right, average length of mitochondria in dorsal and ventral processes of DD neurons of L4 stage wild type and *dve-1(uf171)* mutants. Each dot represents a single mitochondrion. wild type, n=10; *dve-1(uf171)*, n=6. Bars indicate mean ± SEM. ns: not significant, two-way ANOVA with Tukey's multiple comparisons test.

(B) Average intestinal fluorescence intensity measures for wild type, *dve-1(tm4807)*, *dve-1(uf171)* and *dve-1(uf171);ulp-4(lf)* animals expressing *hsp-6pr::GFP*. Bars represent mean ± SEM. One way ANOVA with Tukey's multiple comparisons test. **p <0.01, ****p< 0.0001.

(C) Fluorescent images of transgenic worms expressing the mtUPR reporter *hsp-6pr::*GFP and treated with either empty vector (top) or RNAi targeting *atfs-1* (bottom). *dve-1(uf171)* mutants show increased expression of *hsp-6pr::*GFP under basal conditions compared with control animals and this is reversed by RNAi targeting *atfs-1*. *isp-1* mutants also have elevated mtUPR and are included as a control.

(D) Fluorescent confocal images of iAChR clusters in dorsal nerve cord (DNC) of DD neurons of L4 stage *dve-1(uf171)* mutants treated with either empty vector or RNAi targeting *atfs-1*. *atfs-1* RNAi reverses mtUPR activation in *dve-1* mutants but does not normalize synapse elimination. Scale bar, 5 μm.

(E) Quantitative RT-PCR analysis of predicted DVE-1 targets. Mutation of *dve-1* significantly alters *cul-5*, *spat-3*, and *eel-1* expression, normalized to *act-1* levels. Each point indicates an independent technical replicate. Bars represent mean ± SEM. ***p*<0.001, * *p*<0.05, Welch's t test.

(F) Fluorescent images of transgenic worms expressing the *rpt-3pr::GFP* reporter with or without treatment with the proteasome inhibitor Bortezomib (5 μM).

(G) Quantification of the average number of dendritic spines in L4 stage wild type animals under either control conditions (n=10) or following Bortezomib treatment (n=12). Each dot represents a single animal. Bars represent mean ± SEM.

Supplementary Table 1: DVE-1 ChIP-seq targets

Ubiquitin-Proteasome System DVE-1 ChIP-seq targets			
C. elegans	***H. sapiens***	**Gene Description**	**Enrichment tool**
**Proteasome subunit/composition**			
pas-5	PSMA5	proteasome subunit alpha 5 (20S proteasome)	WormCat, WikiPathways (wormenrichr)
rpt-5	PSMC3	proteasome 26S subunit ATPase 3 (26S proteasome)	WormCat, WikiPathways (wormenrichr)
rpt-6	PSMC5	proteasome 26S subunit ATPase 5 (26S proteasome)	WormCat, WikiPathways (wormenrichr)
**Ubiquitin**			
ubq-1	UBC	Ubiquitin, polyubiquitin locus	WormCat, WikiPathways (wormenrichr)
**E2 Enzyme**			
ubc-2/let-70	UBE2D1/UBE2D2/UBE2D3	E2 ubiquitin conjugating enzyme	WormCat, WikiPathways (wormenrichr)
**E3 Enzyme HECT-Domain**			
wwp-1	ITCH	HECT-domain ubiquitin E3 ligase	WormCat
eel-1	HUWE1	HECT-domain ubiquitin E3 ligase	WormCat
**E3 Enzyme RING-finger complex**			
cul-5	CUL5	RING finger complexe cullin 5	WormCat
rfp-1	RNF20	ring finger protein 20	WormCat
C11H1.3	MGRN1	mahogunin ring finger 1	WormCat
rnf-113	RNF113A	ring finger protein 113A	WormCat
spat-3	RING1/RING2	ring finger protein 1/2	WormCat
**DUB Enzymes**			
usp-48	USP48	Ubiquitin-Specific Protease 48	WormCat
usp-14	USP14	Ubiquitin-Specific Protease 14	WormCat
otub-1	OTUB1/2	otubain-1/2	WormCat
H34C03.2	USP11	Ubiquitin Specific Peptidase 11	WormCat
T22F3.2	USP17L1	Ubiquitin Specific Peptidase 17 Like Family Member 1	WormCat
otub-2	OTUD7A	OTU Deubiquitinase 7A	WormCat
**UPS associated**			
C46F11.6	UBL3	Ubiquitin-like 3	WormCat
wdr-23	DCAF11/WRD23	DDB1 and CUL4 associated factor 11	WormCat
ubql-1	UBQLN4	ubiquilin 4	WormCat
atg-7	ATG7	autophagy related	WormCat
ppm-2	PPM1A	protein phosphatase, Mg2+/Mn2+ dependent 1A	WormCat
K02A6.3		F-box domain	WormCat
spsb-1	SPSB1	a Spry domain-containing socs box protein	WormCat
cpi-2	CST3/6	cystatin C/ cystatin E/M	WormCat
try-6	TMPRSS13	transmembrane serine protease 13	WormCat
spcs-3	SPCS3	signal peptidase complex subunit 3	WormCat
