# Supplementary material for: The homeodomain transcriptional regulator DVE-1 directs a program for synapse elimination during circuit remodeling
Source: Nat Commun. 2023 Nov 18;14:7520. doi: 10.1038/s41467-023-43281-4 (PMC10657367; doi:10.1038/s41467-023-43281-4)
Supplement: Supplementary file 3 — Description of Additional Supplementary Files [file 41467_2023_43281_MOESM3_ESM.pdf]

Description of Additional Supplementary Files

File Name: Supplementary Data 1
Description: Strain list

File Name: Supplementary Data 2
Description: Plasmid list

File Name: Supplementary Data 3
Description: Primer list

File Name: Supplementary Data 4
Description: CRISPR/Cas9 Design

File Name: Supplementary Data 5
Description: ChIP-seq targets and pathway analysis

File Name: Supplementary Data 6
Description: Summary Statistics
